# Targeted metabolomic analysis of amino acid response to L-asparaginase in adherent cells

**DOI:** 10.1007/s11306-014-0634-1

**Published:** 2014-02-07

**Authors:** Preeti Purwaha, Philip L. Lorenzi, Leslie P. Silva, David H. Hawke, John N. Weinstein

**Affiliations:** 1 Department of Bioinformatics and Computational Biology, MD Anderson Cancer Center, University of Texas, Houston, TX 77054 USA; 2 Proteomics Facility, Department of Pathology, MD Anderson Cancer Center, University of Texas, Houston, TX 77054 USA; 3 Department of Systems Biology, MD Anderson Cancer Center, University of Texas, Houston, TX 77054 USA

**Keywords:** L-Asparaginase, Metabolism, Amino acid, Ovarian cancer, Metabolomics, Mass spectrometry

## Abstract

L-asparaginase (L-ASP) is a therapeutic enzyme used clinically for the treatment of childhood acute lymphoblastic leukemia. L-ASP’s anticancer activity is believed to be associated primarily with depletion of asparagine, but secondary glutaminase activity has also been implicated in its anticancer mechanism of action. To investigate the effects of L-ASP on amino acid metabolism, we have developed an LC–MS/MS metabolomics platform for high-throughput quantitation of 29 metabolites, including all 20 proteinogenic amino acids, 6 metabolically related amino acid derivatives (ornithine, citrulline, sarcosine, taurine, hypotaurine, and cystine), and 3 polyamines (putrescince, spermidine, and spermine) in adherent cultured cells. When we examined the response of OVCAR-8 ovarian cancer cells in culture to L-ASP, asparagine was depleted from the medium within seconds. Interestingly, intracellular asparagine was also depleted rapidly, and the mechanism was suggested to involve rapid export of intracellular asparagine followed by rapid conversion to aspartic acid by L-ASP. We also found that L-ASP-induced cell death was more closely associated with glutamine concentration than with asparagine concentration. Time-course analysis revealed the dynamics of amino acid metabolism after feeding cells with fresh medium. Overall, this study provides new insight into L-ASP’s mechanism of action, and the optimized analytical method can be extended, with only slight modification, to other metabolically active amino acids, related compounds, and a range of cultured cell types.

**Electronic supplementary material:**

The online version of this article (doi:10.1007/s11306-014-0634-1) contains supplementary material, which is available to authorized users.

## Introduction

L-asparaginase (L-ASP) is a key therapeutic agent for the treatment of childhood acute lymphoblastic leukemia (ALL). L-ASP catalyzes the deamidation of asparagine and glutamine into aspartic acid and glutamic acid, respectively, releasing ammonia in the process. The resulting amino acid (AA) deprivation induces cell death in tumor cells that lack the ability to synthesize sufficient levels of those AAs (Balasubramanian et al. 2013). Expression of asparagine synthetase (ASNS), which catalyzes the synthesis of asparagine, is negatively correlated with L-ASP’s anticancer activity in leukemia cell lines (Fine et al. 2005; Hutson et al. 1997; Scherf et al. 2000; Su et al. 2008), leukemia patient samples (Leslie et al. 2006), and ovarian cancer cell lines (La Spina et al. 2010; Lorenzi et al. 2006, 2008; Lorenzi and Weinstein 2009). However, asparagine deamidation alone may be insufficient to induce cell death (Asselin et al. 1989; Kitoh et al. 1992), prompting the search for additional metabolites that mediate the cellular response to L-ASP treatment.

In previous studies, we and others have reported a rationale for extending L-ASP treatment to some types of solid tumors (Bussey et al. 2006; Lorenzi et al. 2006, 2008; Scherf et al. 2000; Yu et al. 2012). To further substantiate that rationale, we initiated metabolomics investigations to probe L-ASP’s mechanism of action. Accordingly, we examined the effects of L-ASP on AA metabolism since L-ASP is known to degrade asparagine and glutamine enzymatically. To optimize sample preparation for analysis of AAs in mammalian cells, we examined various conditions for quenching cellular metabolism, extracting metabolites, removing proteins, and normalizing data. We also developed and optimized an LC–MS/MS method to satisfy the following criteria: label-free (i.e., no pre- or post-column derivatization), sufficient chromatographic retention to separate polar AAs, resolution of isotopic and isobaric AAs, short run time, high sensitivity, and large linear dynamic range. We used the optimized protocol to analyze AA metabolism in response to L-ASP treatment in cultured ovarian cancer cells. The protocol can be extended to other mammalian cell types to study the metabolism of AAs and similar compounds.

## Materials and methods

During the course of method development, we analyzed a variety of process parameters, calibration standards, and data normalization strategies. The following materials and methods are based primarily on the optimal set of conditions defined by that process.

### Chemicals and reagents

Standards for cysteine, asparagine, glutamine, tryptophan, ornithine, citrulline, putrescine, spermine, spermidine, sarcosine, taurine, hypotaurine, a mixture containing 17 AAs, an isotopic algal mixture of 16 ^13^C,^15^N-labeled AAs, ^13^C_4_,^15^N_2_-asparagine, ^13^C_4_-asparagine, ^13^C_5_,^15^N_2_-glutamine, ^13^C_3_,^15^N-cysteine, and ^13^C_11_,^15^N_2_-tryptophan were all purchased from Sigma Aldrich (St. Louis, MO). d_8_-putrescine was purchased from CDN isotopes (Quebec, Canada). Pronase and carboxypeptidase Y were purchased from Calbiochem (Darmstadt, Germany). LC/MS-grade water, methanol, and acetonitrile (ACN) were purchased from Burdick and Jackson (Muskegon, MI). Formic acid and heptafluorobutyric acid (HFBA) were obtained from Sigma Aldrich. L-asparaginase was obtained from Lundbeck pharmaceuticals (Deerfield, IL). Medium for cell culture (RPMI 1640) and fetal bovine sera (FBS) were purchased from Thermo HyClone (Logan, Utah). The liquid chromatography column (Zorbax SB C-18; 3.0 × 100 mm, 1.8 μm particle size) was obtained from Agilent (Santa Clara, CA). All other chemicals were of analytical grade and obtained from Sigma.

### Cell culture

OVCAR-8 cells were routinely maintained in RPMI 1640 containing 5 % FBS and 1 % (2 mM) l-glutamine (HyClone). Cultures were grown in an atmosphere of 5 % CO_2_ at 37 °C. For experiments, 120,000 cells were seeded in 10 cm polypropylene dishes for 48 h, then treated with fresh medium containing vehicle, 0.1 U/mL L-ASP (EC_10_), or 0.5 U/mL L-ASP (EC_50_) for 0, 0.01, 8, 24, or 48 h. At the time of treatment (48 h) and time of harvesting the final time point (96 h total), cells were approximately 40 and 80 % confluent, respectively. Zero-time points reflect the untreated state, whereas 0.01 h time points reflect samples that were exposed to the indicated treatment for approximately 30 s and immediately processed as described below. Parallel dishes containing medium without cells were subjected to similar treatments to distinguish between efflux from the cells and degradation processes that occur in the medium. OVCAR-4 cells were also cultured and treated with 0.5 U/mL L-ASP and processed as above.

### Sample preparation

#### General protocol

At *indicated* time points, cells were subjected to washing and extraction of AAs by quenching, addition of extraction solvent, scraping, and disruption of cells for ~30 s using a Disruptor Genie (Scientific Industries Inc.). Samples were then centrifuged at 20,000*g* for 4 min, and supernatants were collected. Cell pellets were saved for DNA analysis for data normalization (Silva et al. 2013). Protein was precipitated and removed by transferring 200 μL of sample to 600 μL of dry ice-cooled 100 % methanol containing internal standards. Precipitated lysates were collected and evaporated to dryness using a SpeedVac (Thermo Scientific). Dried cell lysates were reconstituted in 100 μL mobile phase A. Corresponding samples of medium were processed by combining 200 μL of sample with 600 μL of dry ice-cooled methanol containing internal standards, evaporating to dryness, and reconstituting in 200 μL mobile phase A. Accordingly, final concentrations of cell and medium samples were 2:1 and 1:10, respectively, of the original concentrations.

#### Optimization of quenching

To capture an accurate snapshot of intracellular AA concentrations, it is important to quench metabolism and other cellular processes that could introduce artifacts during the sample preparation process. Building on previous quenching optimization efforts of others (Danielsson et al. 2010; Meinert et al. 2013; Sellick et al. 2011), we compared the quenching abilities of 60 % MeOH containing ammonium bicarbonate (0.85 % w/v) (MeOH/AMBIC), 60 % MeOH containing 70 mM HEPES, and 0.9 % NaCl. Quenching buffer was added directly onto the adherent cells and removed immediately after rinsing the cells twice.

#### Optimization of extraction

AA concentrations measured in cells were compared for extractions performed with multiple combinations of organic solvents, methanol:acetonitrile = 1:1, methanol:chloroform:water = 7:2:1, and acetonitrile:water = 1:1.

### Liquid chromatography and mass spectrometry

Liquid chromatography was performed on an Agilent 1290 Infinity UHPLC system equipped with a Zorbax SB C-18 column (3.0 × 100 mm, 1.8 μm particle size) at 25 °C column temperature. The mobile phase consisted of (A) water and (B) acetonitrile, each containing 0.3 % HFBA and 0.5 % formic acid. Gradient conditions were: 0.01 to 2 min = 2 to 30 % B; 2 to 4.1 min = 30 to 40.0 % B; 4.1 to 4.8 min = 40 to 45 % B; 4.8 to 4.9 min = 45 to 90 % B; 4.9 to 5.5 min = 90 % B; 5.5 to 5.6 min = 90 to 2 % B; and 5.6 to 8.0 min = 2 % B. Injection volume was 5.0 μL and flow rate was 0.3 mL/min. MS/MS analysis was performed on an Agilent 6460 triple quadrupole equipped with a jet stream electrospray ionization (ESI) source. Multiple reaction monitoring (MRM) was performed in the positive ion mode. Other MS parameters included: gas temperature at 300 °C, drying gas at 7 L/min, nebulizer pressure at 50 psi, sheath gas temperature at 325 °C, sheath gas flow at 10 L/min, capillary voltage at 3750 V, and nozzle voltage at 0 V. Source conditions were optimized for each AA by the Mass Hunter Optimizer B.04.01. The MRM transitions and optimized parameters (e.g., collision energy, fragmentor voltage, and dwell time) used for each AA are summarized in Supplementary Table 1.

#### Standards and internal standards

A mixture of 17 AA standards was used for initial assay development. Standards for cysteine, asparagine, glutamine, tryptophan, ornithine, citrulline, putrescine, spermine, spermidine, sarcosine, taurine, and hypotaurine were combined with the mixture of 17 AAs for construction of calibration curves and optimization of MS parameters. Calibration standard mixes were prepared in mobile phase A at concentrations ranging from 1 nM to 5 mM. An internal standard mixture was made by combining an algal hydrolysate containing 16 isotopic ^13^C, ^15^N-labeled AAs with ^13^C_4_, ^15^N_2_-asparagine, ^13^C_5_, ^15^N_2_-glutamine, ^13^C_3_, ^15^N-cysteine, ^13^C_11_, ^15^N_2_-tryptophan, and d_8_-putrescine. Optimal concentrations of internal standards were found to be 100 μg/mL of algal mix and 10 μM each of labeled asparagine, glutamine, cysteine, tryptophan, and putrescine. The individual AA concentrations in the algal mix are reported in Supplementary Table 3. Optimal fragmentor voltage, collision energy, and MRM transitions were determined for labeled and unlabeled AAs (Supplementary Tables 1 and 2).

#### LC/MS/MS data acquisition and analysis

Mass Hunter (version B.04.01) was used for data acquisition. Mass Hunter Qualitative Analysis and Quantitative Analysis were used for data processing. The most abundant MRM transition was selected for each analyte (Supplementary Table 1). The area under the curve for each analyte of interest and its corresponding isotopic internal standard was obtained from the analyte’s MRM transition and retention time. The ratio of those areas was further normalized by DNA concentration (a surrogate for cell number).

#### Method validation

Calibration standards were analyzed in triplicate for each unlabeled compound to determine retention times, limits of detection (LOD), limits of quantitation (LOQ), coefficient of regression (R^2^), and dynamic range. Calibration curves created for each analyte were fitted using linear regression. To address assay reproducibility, calibration standards were analyzed in triplicate on three different days. The coefficient of variation (%CV) was calculated at each concentration within the linear range of the assay.

## Results and discussion

### Sample preparation

#### Optimization of quenching

**Fig. 1:**
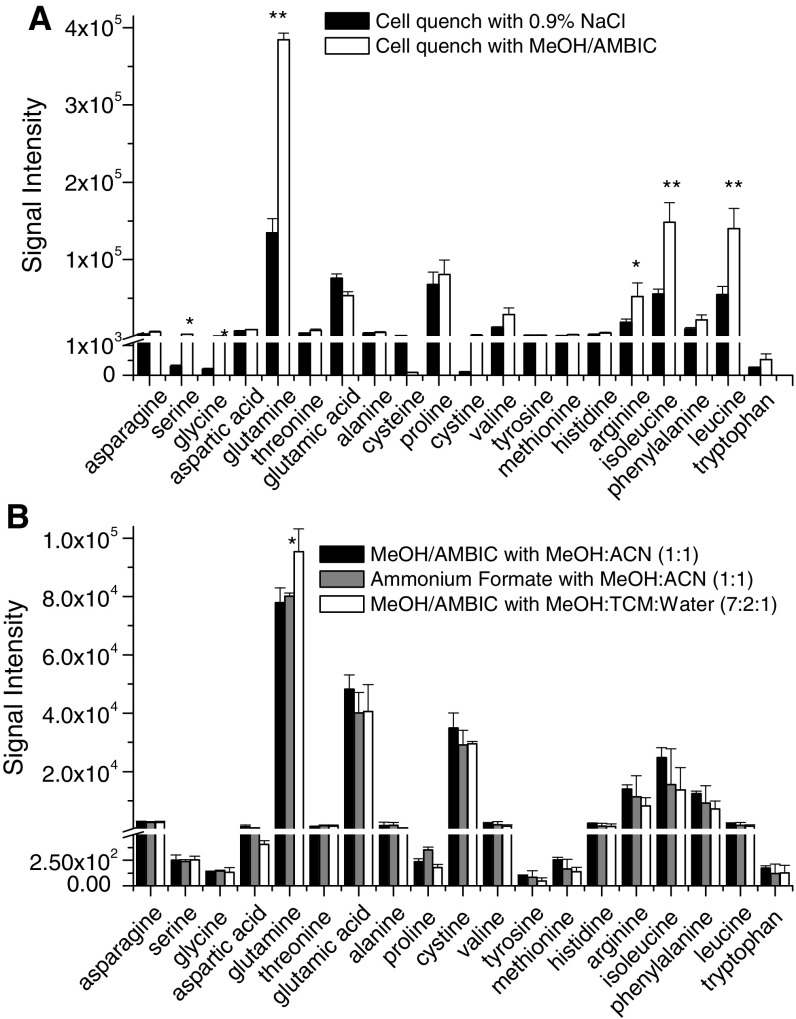
Optimization of sample preparation protocol. **a** Comparison of metabolism quenching by dry ice-cooled (−40 °C) MeOH/AMBIC and ice-cooled 0.9 % NaCl. Quenching buffer was added directly onto OVCAR-8 cells and removed immediately after rinsing the cells twice. **b** Comparison of solvent combinations for metabolite quenching and extraction: MeOH/AMBIC with MeOH/ACN (1:1), ammonium formate with MeOH/ACN (1:1) versus MeOH/AMBIC with MeOH/chloroform/water (7:2:1). *Error bars* represent standard error of the mean for three biological replicates, one-way ANOVA, *p ≤ 0.05, **p ≤ 0.01

Sufficiently rapid quenching of metabolism is a major requirement for the measurement of metabolites in cells or tissues because the half-lives of some metabolically active compounds can be just a few seconds (Dietmair et al. 2010; Kronthaler et al. 2012; Sellick et al. 2009; Tran et al. 2012) The ideal quenching solution should stop metabolism immediately and also reduce leakage of intracellular metabolites (Kronthaler et al. 2012). For that purpose, rapid quenching with cold organic solvents has been used previously (De Jonge et al. 2012; Kronthaler et al. 2012; Lorenz et al. 2011; Sellick et al. 2009; Tran et al. 2012). Quenching with ice-cooled 0.9 % NaCl or dry ice-cooled methanol (60 %) containing 0.85 % ammonium bicarbonate (MeOH/AMBIC) has been reported to be effective (Kronthaler et al. 2012; Sellick et al. 2009; Tran et al. 2012). On the basis of those previous reports, we compared quenching by dry ice-cooled MeOH/AMBIC and ice-cooled 0.9 % NaCl. The efficiency of quenching was assessed on the basis of intracellular AA concentrations, which reflect both metabolic and transport processes. Quenching cells with MeOH/AMBIC resulted in 1.7- to 10-fold greater intracellular AA concentrations than did quenching with 0.9 % NaCl (Fig. 1a). Therefore, cells were washed with MeOH/AMBIC to quench metabolism. Substitution of 70 mM HEPES for AMBIC yielded no significant increase in intracellular signal intensity (data not shown).

#### Optimization of extraction

The next sample preparation step to be optimized was extraction of AAs from cells. We compared three different combinations of quenching and extraction: quenching with MeOH/AMBIC followed by extraction with MeOH/ACN (1:1) (Dietmair et al. 2010); quenching with MeOH containing 70 mM HEPES followed by extraction with MeOH/ACN (1:1) (Kronthaler et al. 2012), and quenching with MeOH/AMBIC followed by extraction with MeOH/chloroform/water (7:2:1) (Bi et al. 2013). Although we saw no significant differences among the quenching/extraction combinations evaluated, we selected MeOH/AMBIC quenching followed by MeOH/chloroform/water (7:2:1) extraction because it yielded the greatest signal intensity for the metabolically important AA glutamine (Fig. 1b).

Sample preparation was further optimized by comparing cell disruption and protein precipitation conditions. When we compared no cell disruption with cell disruption for 30 s, cell disruption yielded 1.5- to 2.0-fold greater signal intensity across the 29 analytes (data not shown). Next, we compared protein precipitation using just 100 % methanol or acetonitrile (without a filter plate) with protein precipitation using 10 different protein precipitation/filtration plates. The signal intensity for most metabolites was greatest when we used the Phenomenex Strata plate (Phenomenex, Torrance, CA) (data not shown). The filtrates were evaporated to dryness at room temperature using a SpeedVac, stored as dry samples at −80 °C, and reconstituted with mobile phase A at the time of analysis. Final concentrations of the cell and medium samples were 2:1 and 1:10, respectively, of the original concentrations.

### LC–MS/MS method development and validation

**Fig. 2:**
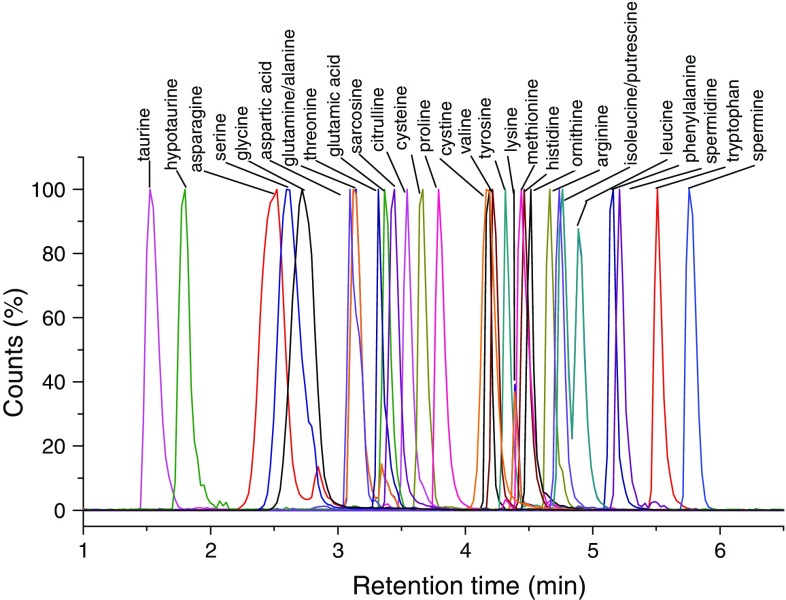
MRM transitions of all 29 metabolites (200 μM). Liquid chromatography was performed on an Agilent 1290 Infinity UHPLC system equipped with a Zorbax SB C-18 column (3.0 × 100 mm, 1.8 μm) at 25 °C column temperature. The mobile phases consisted of water and acetonitrile each containing 0.3 % HFBA and 0.5 % formic acid

Our first goal was to develop a reverse-phase chromatography method capable of retaining polar AAs beyond the void volume without the need for pre- or post-column derivatization. That was achieved using ion-pairing with heptafluorobutyric acid (HFBA) (Fig. 2). Notably, HFBA reduced ion suppression for most of the polar AAs (data not shown) and also increased the chromatographic separation between isomeric compound pairs (e.g., leucine/isoleucine) and between isobaric compound pairs (e.g., glutamine/lysine) (Supplementary Figure 1). Figure 2 illustrates the final chromatographic separation achieved for the 29 metabolites. Optimal MRM transitions, fragmentor voltage, collision energy, and cell accelerator voltage were identified for each unlabeled and isotopically labeled compound in positive ion mode (Supplementary Tables 1 and 2). To improve assay performance and prevent MS source contamination, the first minute of post-column eluent was diverted to waste. The method was validated by analyzing calibration standards in triplicate for each unlabeled compound to determine linear dynamic range, R^2^, retention time, limit of detection (LOD), and limit of quantitation (LOQ) (Table 1). LODs ranged from 0.002 (for alanine) to 0.250 μM (for asparagine), LOQs ranged from 0.007 to 0.760 μM, and the linear dynamic range was two to five log units. Inter- and intra-day reproducibility of retention time was also excellent with %CVs ranging from 0 to 0.47 %.

**Table 1:** LC-MS/MS method development and validation

AA	Linear dynamic range (LLOQ^a^–HLOQ^b^)	R^2^	Retention time (min)	LOD (μM)	LOQ (μM)
Alanine	7.3 nM–750 μM	0.992	3.11	0.002	0.007
Arginine	16.0 nM–750 μM	0.989	4.67	0.006	0.016
Asparagine	0.76 μM–500 μM	0.998	2.52	0.250	0.760
Aspartic acid	0.45 μM–375 μM	0.998	3.09	0.150	0.450
Citrulline	7.0 nM–250 μM	0.989	3.55	0.002	0.007
Cysteine	0.23 μM–250 μM	0.981	3.66	0.076	0.230
Cystine	66.0 nM–312.5 μM	0.999	4.16	0.022	0.066
Glutamic acid	66.0 nM–750 μM	0.992	3.37	0.022	0.066
Glutamine	49.0 nM–500 μM	0.996	3.13	0.016	0.049
Glycine	0.14 μM–375 μM	0.989	2.72	0.049	0.140
Histidine	99.0 nM–750 μM	0.995	4.47	0.033	0.099
Hypotaurine	0.67 μM–5,000 μM	0.990	1.81	0.220	0.670
Isoleucine	27.0 nM–375 μM	0.988	4.74	0.009	0.027
Leucine	70.0 nM–750 μM	0.983	4.89	0.023	0.070
Lysine	75.0 nM–375 μM	0.996	4.39	0.025	0.075
Methionine	0.44 μM–750 μM	0.993	4.44	0.144	0.440
Ornithine	0.18 μM–500 μM	0.995	4.52	0.059	0.180
Phenylalanine	0.10 μM–750 μM	0.991	5.16	0.033	0.100
Proline	80.0 nM–375 μM	0.993	3.79	0.026	0.080
Putrescine	1.42 μM–250 μM	0.978	4.77	0.470	1.420
Sarcosine	0.10 μM–250 μM	0.990	3.44	0.033	0.100
Serine	0.20 μM–750 μM	0.983	2.60	0.070	0.200
Spermidine	4.26 μM–250 μM	0.983	5.21	1.410	4.260
Spermine	53.0 nM–250 μM	0.991	5.76	0.017	0.053
Taurine	67.0 nM–150 μM	0.980	1.51	0.022	0.067
Threonine	22.0 nM–750 μM	0.996	3.32	0.007	0.022
Tryptophan	0.39 μM–500 μM	1.000	5.51	0.130	0.390
Tyrosine	0.47 μM–750 μM	0.992	4.31	0.150	0.470
Valine	48.0 nM–750 μM	0.984	4.19	0.016	0.048

External calibration standards were analyzed for each compound to determine dynamic range, R^2^, retention time, LOD, and LOQ of unlabeled compounds

^a^Lower limit of quantitation

^b^Higher limit of quantitation

The use of stable isotope-labeled AAs as internal standards enabled us to correct for drift in instrument performance and for variation that arose downstream of sample precipitation for all individual AAs. All labeled AAs co-eluted with their non-labeled counterparts. In total, 29 external AA standards and 21 isotope-labeled internal standards were included in the 8-min assay. Sarcosine was normalized using ^13^C_2_, ^15^N-glycine as the internal standard. Polyamines, such as spermine and spermidine, for which no isotopically labeled analogs were available, were normalized using d_8_-putrescine as the internal standard. Cystine (Cys-Cys dipeptide) was normalized using ^13^C_3_, ^15^N-cysteine as the internal standard. Taurine, hypotaurine, ornithine, and citrulline were normalized using ^13^C_4_, ^15^N_2_-asparagine as the internal standard. In addition to the comprehensive inclusion of internal standards for accurate quantitation, specific advantages of the LC–MS/MS method include: no requirement for derivatization, improved chromatographic retention of polar AAs, resolution of isotopic and isobaric AAs, short run time, and, on the basis of those improvements, greater measurement accuracy, sensitivity, and linear dynamic range.

While evaluating and validating the LC–MS/MS method, we observed minor glutamine degradation that was determined to be due to cyclization to pyroglutamic acid in the ESI ion source. A separate manuscript (Purwaha et al. manuscript in preparation) characterizes that reaction. Of note, we found that using ^13^C, ^15^N-glutamine as an internal standard serves as a basis for data correction, accounting for in-source conversion of glutamine to pyroglutamic acid.

### Amino acid metabolism in ovarian cancer cells treated with L-ASP

**Fig. 3:**
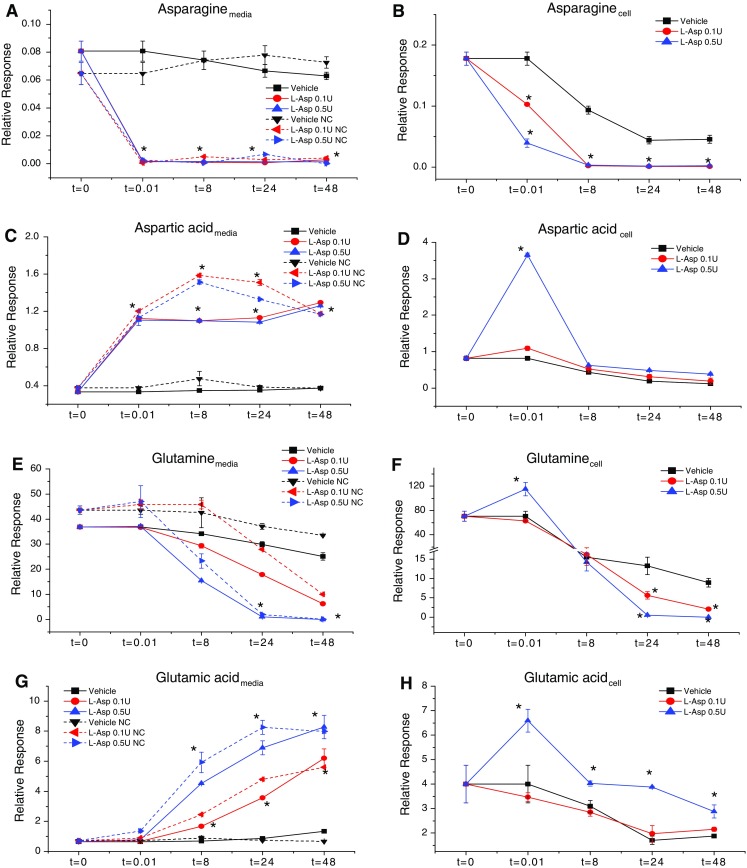
Time course analysis of the four canonical AAs involved directly in enzymatic activity of L-ASP. OVCAR-8 cells and medium (after incubation with cells or no cells (“NC”)) were collected after treatment with vehicle, low dose (0.1 U/mL), or high dose (0.5 U/mL) of L-ASP for 0, 0.01, 8, 24, or 48 h. Concentration of asparagine (**a**, **b**), aspartic acid (**c**, **d**), glutamine (**e**, **f**), and glutamic acid (**g**, **h**) in medium and cell lysates, respectively. Cell lysate data were normalized to DNA concentration of the corresponding cell pellet. *Error bars* represent standard error of the mean for three biological replicates, paired *t* test, *p ≤ 0.05. The spikes in intensity for cells were reproducible in multiple experiments (see text)

**Fig. 4:**
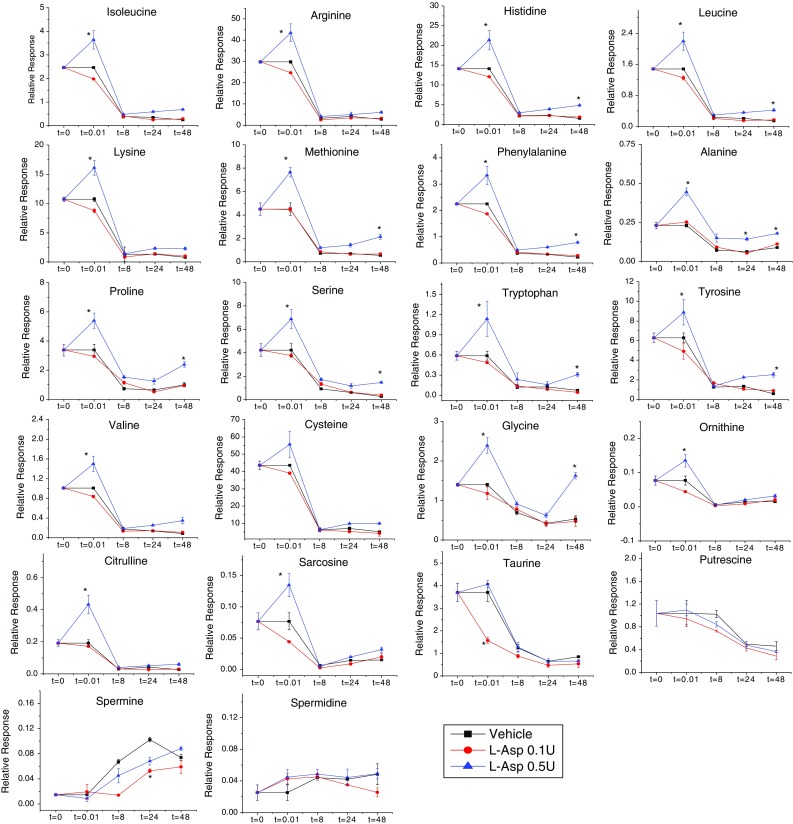
Time course analysis of AAs and derivatives in OVCAR-8 cells collected after treatment with vehicle, low dose L-ASP (0.1 U/mL), or high dose L-ASP (0.5 U/mL) for 0, 0.01, 8, 24, or 48 h. Data were normalized to DNA concentration of the corresponding cell pellet. *Error bars* represent standard error of the mean for three biological replicates, paired *t* test, *p ≤ 0.05. Two metabolites not represented in the figure were found to be below the limit of detection

**Fig. 5:**
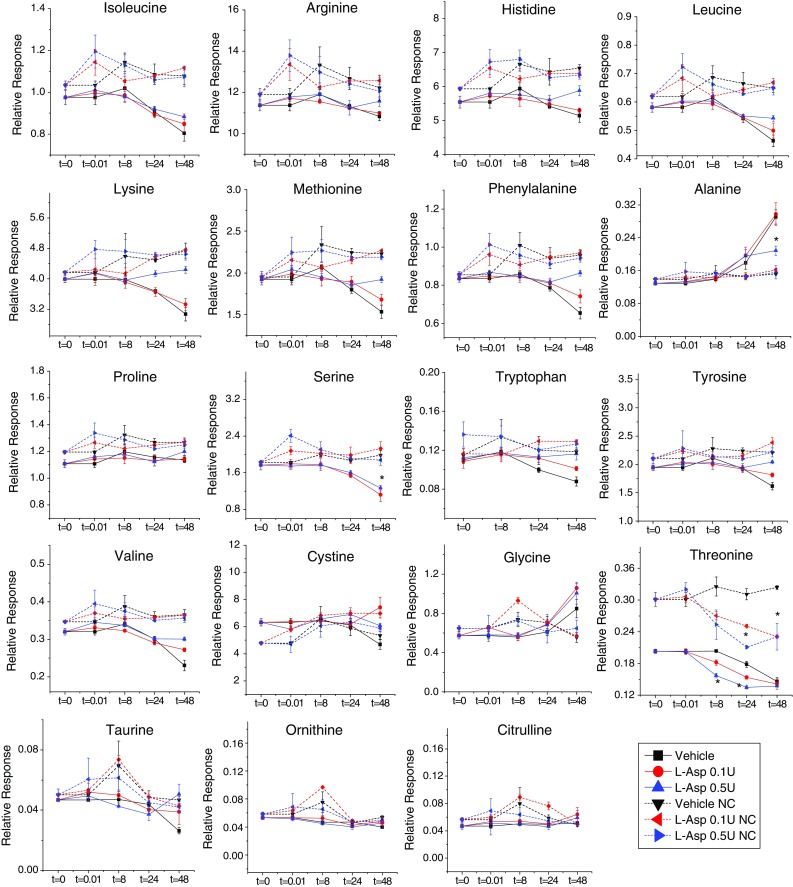
Time course analysis of AAs and their derivatives in medium incubated with OVCAR-8 cells or without cells (“NC”) in vehicle, in low dose L-ASP (0.1 U/mL), or in high dose L-ASP (0.5 U/mL) L-ASP for 0, 0.01, 8, 24, or 48 h. *Error bars* are standard error of the mean for three biological replicates, paired *t* test, *p ≤ 0.05. Six metabolites not represented in the figure were found to be below the limit of detection

We used our optimized assay to conduct a time course analysis of AA concentrations in response to L-ASP treatment of ovarian cancer cells and gained new insight into the drug’s mechanism(s) of action (Figs. 3, 4, 5, and Supplementary Fig 2). First, both low (0.1 U/mL) and high (0.5 U/mL) doses of L-ASP fully depleted asparagine within seconds in the medium (with or without cells present) and within 8 h in OVCAR-8 cells (Fig. 3a, b). Those observations prompted the question: since L-ASP cannot penetrate cells, what is the mechanism by which intracellular asparagine is depleted in response to L-ASP?

To address that question, we first tested the hypothesis that L-ASP causes an increase in the rate of incorporation of intracellular asparagine into cellular protein. Total protein was extracted from OVCAR-8 cells treated with or without L-ASP (0.5 U/mL) and subsequently hydrolyzed using proteases to yield free amino acids (Sweeney and Walker 1993). Comparison of the asparagine levels between the two groups showed no differences between vehicle- and L-ASP-treated cells (data not shown). Hence, the mechanism of intracellular asparagine depletion in response to L-ASP does not appear to involve increased rate of incorporation into cellular protein.

To further investigate how intracellular asparagine is depleted in response to L-ASP treatment, we next pre-treated OVCAR-8 cells with ^13^C_4_-labeled asparagine to test the hypothesis that asparagine is rapidly exported from cells in response to L-ASP treatment. After 30 min of incubation with medium in which the usual ^12^C_4_-asparagine was replaced with ^13^C_4_-asparagine (50 mg/L), ^13^C_4_-asparagine comprised approximately 90 % of the total intracellular asparagine. The cells were then treated for ~30 s with fresh medium containing ^12^C_4_-asparagine and 0.5 U/mL L-ASP. In that short time frame, extracellular ^13^C_4_-aspartic acid increased from 0 to about 40 % of the total aspartic acid content (data not shown) possibly either by export of ^13^C_4_-aspartic from the intracellular compartment or by first exporting ^13^C_4_-asparagine followed by L-ASP-mediated degradation of asparagine to aspartic acid in the extracellular compartment. Given that L-ASP completely depleted extracellular asparagine within 30 s (Fig. 3a), it seems reasonable to speculate that L-ASP causes a strong, extracellularly-directed asparagine gradient that leads to asparagine efflux from the intracellular compartment followed by L-ASP-mediated degradation to aspartic acid in the extracellular compartment. Hence, the mechanism by which L-ASP depletes intracellular asparagine appears to involve rapid export of intracellular asparagine to the outside of the cell, where it is rapidly converted to aspartic acid by L-ASP.

To assess whether the 0.5 U/mL L-ASP-induced increase in intracellular aspartic acid concentration was derived from asparagine, we next pre-treated OVCAR-8 cells with ^13^C_4_-asparagine for 30 min followed by vehicle or L-ASP (0.5 U/mL) treatment. The resulting time course (with measurements at 0, 0.01, 0.5, and 1 h) indicated that within 30 s of L-ASP treatment, extracellular ^13^C_4_-asparagine was fully converted into ^13^C_4_-aspartic acid (Supplementary Figure 3A). Intracellular ^13^C_4_-asparagine decreased from 90 to 44 % of the total (^12^C + ^13^C), and intracellular ^13^C_4_-aspartic acid increased from 50 to 75 %, suggesting that 25 % of the latter L-ASP-induced increase in ^13^C_4_-aspartic acid was derived from intracellular asparagine (Supplementary Figure 3B). These findings suggest that the rapid increase in intracellular aspartic acid is derived in part from intracellular asparagine, but most of it may be derived from the rapid uptake of extracellular aspartic acid.

The observation of rapid asparagine depletion (Fig. 3a, b) was unexpected primarily because 0.1 and 0.5 U/mL L-ASP were previously noted to cause approximately 10 and 50 % cell death, respectively, after 48 h (Lorenzi et al. 2006, 2008). That inconsistency between the timing of metabolite depletion and the overall drug response prompted the question: which other metabolites might be associated with L-ASP-induced cell death? Interestingly, depletion of glutamine in both the extracellular compartment (culture medium) and intracellular compartment (cell pellet) reached a maximum at 24 h, which was sustained through 48 h (Fig. 3e, f), suggesting that glutamine is closely associated with cell death in response to L-ASP treatment in OVCAR-8 cells. Notably, glutamic acid concentrations were elevated across the entire time series following treatment with an EC_50_ dose (0.5 U/mL) of L-ASP, whereas glutamic acid concentrations following an EC_10_ dose (0.1 U/mL) were unchanged relative to the vehicle control. To further probe whether the elevated glutamic acid concentrations played a role in cell death by the EC_50_ dose (0.5 U/mL) of L-ASP, glutamic acid was tested for anticancer activity toward OVCAR-8 cells but had no effect on cell proliferation (as measured by CellTiter Blue assay; data not shown).

Another unexpected feature of the AA response to treatment with high dose (0.5 U/mL) L-ASP was an immediate increase in the intracellular concentrations of all AAs except asparagine, as shown by the upward spike at the 0.01 h time point (Figs. 3, 4). That phenomenon has been repeated in multiple, independent follow-up experiments by multiple investigators in our laboratory (data not shown). We initially hypothesized that 0.5 U/mL L-ASP rapidly up-regulates AA concentrations through the induction of autophagy. However, additional experiments conducted using wild-type and ATG5 (a vital autophagy regulatory gene)-knockout mouse embryonic fibroblasts (MEFs) showed that the initial increase of intracellular amino acid concentration with L-ASP treatment was observed irrespective of autophagy status, suggesting that autophagy may not be involved in the rapid metabolite increase.

Two additional features of the observed AA responses to fresh medium (vehicle) and L-ASP are worth further discussion. First, after routine feeding of OVCAR-8 cells with fresh medium, the intracellular concentrations of most AAs were depleted within 8 h, and all AAs were generally depleted by 24 h (Figs. 3, 4). Those trends were presumably due to metabolic consumption, since AAs are needed to maintain cancer cell growth and proliferation. Consistent with those observations, the polyamine putrescine was converted into spermine (a higher order polyamine) over 24 h, suggesting anabolic behavior of polyamines in OVCAR-8 cells.

Second, although the majority of extracellular AA concentrations remained constant, Fig. 5 illustrates that the concentrations of several AAs decreased in the medium over time, suggesting that they were consumed by the proliferating OVCAR-8 cells, particularly at the 48 h time point. Only one AA, alanine, was apparently exported into the medium during that timeframe. The AA threonine showed a dose-dependent depletion in medium (Fig. 5) with L-ASP treatment but this phenomena was undetectable in the cell lysates. Threonine levels were also lower in medium incubated with cells in comparison to medium incubated without cells, suggesting that cells take up and/or metabolize threonine almost instantaneously.

## Concluding remarks

We describe here the development and application of a targeted LC–MS/MS metabolomics platform for high-throughput quantitation of free AAs and related metabolites to study the mechanism of action of L-ASP. The study revealed that inhibition of cell metabolism by L-ASP was more closely associated with glutamine concentration than asparagine concentration. The platform and optimized method described here can be extended to include measurement of other metabolically active AAs and related compounds in adherent cell lines or, with slight modification, in suspension cell lines.

## Electronic supplementary material

Below is the link to the electronic supplementary material.

Supplementary material 1 (DOCX 244 kb)
