# Supplementary material for: Targeted metabolomic analysis of amino acid response to L-asparaginase in adherent cells
Source: Metabolomics. 2014 Feb 7;10(5):909–19. doi: 10.1007/s11306-014-0634-1 (PMC4145215; doi:10.1007/s11306-014-0634-1)
Supplement: Supplementary file 1 — Supplementary material 1 (DOCX 244 kb) [file 11306_2014_634_MOESM1_ESM.docx]

**Targeted metabolomic analysis of amino acid response to L-asparaginase in adherent cells**

Preeti Purwaha^1*^, Philip L. Lorenzi^1*^, Leslie P. Silva^1^, David H. Hawke^2^, and John N. Weinstein^1,3^

^1^Department of Bioinformatics and Computational Biology and ^2^Proteomics Facility, Department of Pathology, MD Anderson Cancer Center, University of Texas, Houston, TX 77054

^3^Department of Systems Biology, MD Anderson Cancer Center, University of Texas, Houston, TX 77054.

*These authors contributed equally.

*Corresponding author

Philip L. Lorenzi: [pllorenzi@mdanderson.org](mailto:pllorenzi@mdanderson.org)

Phone: 713-792-9999

Fax: 713-563-4235

Abbreviated title: Amino acid response to L-asparaginase

**Supplementary**

**Fig 1.** MRM transitions of isobaric and isomeric compounds showing chromatographic separation of (A) glutamine and lysine and (B) isoleucine and leucine.

**Table 1**. Optimized MRM transitions, dwell times, fragmentor voltage, collision energy, and cell accelerator voltage of unlabeled compounds.

| **^*^Compound** | **Precursor Ion >**  **Product Ion**  **(m/z)** | **Dwell**  **Time (ms)** | **Fragmentor Voltage**  **(V)** | **Collision Energy (V)** | **Accelerator Voltage**  **(V)** |
| --- | --- | --- | --- | --- | --- |
| cystine | 241.2 > 151.9 | 10 | 86 | 9 | 7 |
| tryptophan | 205 > 188 | 10 | 80 | 15 | 7 |
| spermine | 203 > 129 | 15 | 104 | 18 | 7 |
| tyrosine | 182.2 > 136 | 10 | 52 | 9 | 7 |
| citrulline | 176.11 > 70 | 15 | 78 | 10 | 7 |
| arginine | 175.2 > 70 | 15 | 104 | 22 | 7 |
| phenylalanine | 166.1 > 119.9 | 15 | 78 | 14 | 7 |
| histidine | 156.1 > 109.9 | 15 | 76 | 13 | 7 |
| methionine | 150.1 > 103.9 | 15 | 80 | 6 | 7 |
| glutamic acid | 148.06 > 84 | 15 | 74 | 14 | 7 |
| glutamine | 147.1 > 84 | 15 | 76 | 10 | 7 |
| lysine | 146.9 > 83.9 | 15 | 78 | 15 | 7 |
| spermidine | 146 > 129 | 15 | 104 | 18 | 7 |
| aspartic acid | 134.05 > 74 | 15 | 80 | 10 | 7 |
| ornithine | 133.1 > 70.1 | 15 | 78 | 10 | 7 |
| asparagine | 133.1 > 87.1 | 15 | 74 | 10 | 7 |
| leucine | 132.1 > 85.8 | 20 | 80 | 10 | 7 |
| isoleucine | 132.1 > 85.8 | 20 | 76 | 10 | 7 |
| taurine | 126.02 > 44.2 | 15 | 104 | 18 | 7 |
| cysteine | 122 > 76 | 15 | 80 | 15 | 7 |
| threonine | 120 > 73.9 | 18 | 76 | 6 | 7 |
| valine | 117.8 > 71.9 | 15 | 76 | 7 | 7 |
| proline | 115.8 > 69.9 | 15 | 78 | 17 | 7 |
| hypotaurine | 110 > 30.1 | 15 | 104 | 18 | 7 |
| serine | 106 > 59.9 | 15 | 78 | 9 | 7 |
| alanine | 90 > 43.9 | 15 | 80 | 10 | 7 |
| sarcosine | 89.9 > 44 | 15 | 104 | 18 | 7 |
| putrescine | 89 > 72 | 15 | 104 | 18 | 7 |
| glycine | 76 > 30 | 15 | 76 | 4 | 7 |

^*^All listed compounds are L-isomers, where pertinent.

**Table 2**. Optimized MRM transitions, dwell times, fragmentor voltage, collision energy and cell accelerator voltage of labeled compounds used as internal standards.

| **Internal standard** | **Precursor Ion > Product Ion**  **(m/z)** | **Dwell time (ms)** | **Fragmentor Voltage**  **(V)** | **Collision Energy (V)** | **Accelerator Voltage**  **(V)** |
| --- | --- | --- | --- | --- | --- |
| ^13^C^15^N typtophan | 218.24 > 156.1; | 15 | 86 | 14 | 7 |
| ^13^C^15^N tyrosine | 192.2 > 98.1 | 15 | 86 | 30 | 7 |
| ^13^C^15^N arginine | 185.2 > 75.1 | 15 | 102 | 30 | 7 |
| ^13^C^15^N phenylalanine | 176.2 > 129.1 | 15 | 86 | 10 | 7 |
| ^13^C^15^N histidine | 165.2 > 118 | 15 | 48 | 14 | 7 |
| ^13^C^15^N methionine | 156.2 > 60.1 | 15 | 52 | 18 | 7 |
| ^13^C^15^N glutamine | 154.2 > 89.1 | 15 | 78 | 10 | 7 |
| ^13^C^15^N lysine | 155.2 > 90.1 | 15 | 84 | 18 | 7 |
| ^13^C^15^N asparagine | 139.06 > 92.1; | 15 | 78 | 10 | 7 |
| ^13^C^15^N leucine | 139.2 > 92.1 | 15 | 58 | 10 | 7 |
| ^13^C^15^N aspartic acid | 139.05 > 77 | 15 | 78 | 10 | 7 |
| ^13^C^15^N glutamic acid | 154.06 > 89 | 15 | 80 | 10 | 7 |
| ^13^C^15^N isoleucine | 139.2 > 92.1 | 15 | 50 | 10 | 7 |
| ^13^C^15^N cysteine | 126.14 > 61.1 | 15 | 72 | 14 | 7 |
| ^13^C^15^N threonine | 125.1 > 78.1 | 15 | 74 | 10 | 7 |
| ^13^C^15^N valine | 124 > 77.1 | 15 | 80 | 10 | 7 |
| ^13^C^15^N proline | 122 > 75.1 | 15 | 88 | 18 | 7 |
| ^13^C^15^N serine | 110 > 63.1 | 15 | 72 | 10 | 7 |
| ^13^C^15^N alanine | 94 > 47.1 | 15 | 44 | 10 | 7 |
| ^13^C^15^N glycine | 79 > 32.2 | 15 | 48 | 10 | 7 |
| putrescine-d_8_ | 97.2>80.2 | 15 | 106 | 10 | 7 |

**Table 3**. Concentrations of isotopic AAs in the algal mixture.

| **Isotopic standard** | **Conc. in 1 mg/mL algal mix (mM)** | **Rel. Mol %**  **(Sigma)** |
| --- | --- | --- |
| ^13^C^15^N serine | 3.1 ± 0.26 | 4.4 |
| ^13^C^15^N glycine | 5.89 ± 1.1 | 10.8 |
| ^13^C^15^N alanine | 11.06 ± 1.82 | 14 |
| ^13^C^15^N proline | 3.88 ± 0.48 | 4.7 |
| ^13^C^15^N valine | 13.38 ± 1.55 | 8.1 |
| ^13^C^15^N tyrosine | 0.376 ± 0.036 | 0.8 |
| ^13^C^15^N lysine | 1.21 ± 0.15 | 3.9 |
| ^13^C^15^N methionine | 0.98 ± 0.11 | 2.7 |
| ^13^C^15^N histidine | 0.25 ± 0.022 | 0.9 |
| ^13^C^15^N arginine | 1.56 ±0.30 | 2.9 |
| ^13^C^15^N leucine | 15.35 ± 1.42 | 9.6 |
| ^13^C^15^N isoleucine | 8.17 ± 0.87 | 5.8 |
| ^13^C^15^N phenylalanine | 3.45 ± 0.824 | 4.4 |
| ^13^C^15^N threonine | 8.61 ± 0.28 | 6.2 |
| ^13^C^15^N aspartic acid | 9.52±0.97 | 10.2 |
| ^13^C^15^N glutamic acid | 14.30 ± 1.53 | 10.1 |

**Fig 2.** Time course analysis of the four canonical AAs involved directly in enzymatic activity of L-ASP. OVCAR-4 cells were collected after treatment with vehicle, or 0.5 U/mL of L-ASP for 0, 0.01, 8, or 24 hours. Concentration of asparagine (A, B), aspartic acid (C, D), glutamine (E, F), and glutamic acid (G, H) in culture medium and cell lysates, respectively. Cell lysate data were normalized to DNA concentration of the corresponding cell pellet. Error bars represent standard error of the mean for three biological replicates, paired t-test, *p ≤ 0.05.

**Fig 3.** ^13^C_4_-asparagine in culture medium was used to study the flux of asparagine with or without L-ASP treatment in culture medium and cell lysates. OVCAR-8 cells were pretreated with medium containing ^13^C_4_-asparagine for 30 min followed by vehicle or L-ASP (0.5 U/mL) treatment for 0.01, 0.5 and 1h. Time course analysis of (A) extracellular ^13^C_4_- aspartic acid, and (B) intracellular ^13^C_4_-asparagine and ^13^C_4_-aspartic acid after vehicle or L-ASP treatment.

**Table 4**. IDs (HMDB, CAS and ChEBI) of metabolites.

| **Compound** |  | **IDs** |  |
| --- | --- | --- | --- |
|  | **HMDB** | **CAS** | **ChEBI** |
| alanine | HMDB00161 | 56-41-7 | amino acid zwitterion |
| arginine | HMDB00517 | 74-79-3 | L-alpha-amino acid |
| asparagine | HMDB00168 | 70-47-3 | D-alpha-amino acid |
| aspartic acid | HMDB00191 | 56-84-8 | L-alpha-amino acid |
| citrulline | HMDB00904 | 372-75-8 | aliphatic acyclic compounds |
| cysteine | HMDB00574 | 52-90-4 | cysteine zwitterion |
| cystine | HMDB00192 | 56-89-3 | amino acid zwitterion |
| glutamic acid | HMDB00148 | 56-86-0 | alpha-amino acid |
| glutamine | HMDB00641 | 56-85-9 | alpha-amino acid |
| glycine | HMDB00123 | 56-40-6 | amino acid zwitterion |
| histidine | HMDB00177 | 71-00-1 | alpha-amino acid |
| hypotaurine | HMDB00965 | 300-84-5 | zwitterion |
| isoleucine | HMDB00172 | 73-32-5 | alpha-amino acid |
| leucine | HMDB00687 | 61-90-5 | alpha-amino acid |
| lysine | HMDB00182 | 56-87-1 | L-alpha-amino acid |
| methionine | HMDB00696 | 63-68-3 | amino acid zwitterion |
| ornithine | HMDB00214 | 70-26-8 | alpha-amino acid |
| phenylalanine | HMDB00159 | 63-91-2 | aromatic amino acid |
| proline | HMDB00162 | 147-85-3 | amino acid zwitterion |
| putrescine | HMDB01414 | 110-60-1 | alkane-alpha, omega-diamine |
| sarcosine | HMDB00271 | 107-97-1 | amino acid zwitterion |
| serine | HMDB00187 | 56-45-1 | amino acid zwitterion |
| spermidine | HMDB01257 | 124-20-9 | triamine |
| spermine | HMDB01256 | 71-44-3 | tetraamine |
| taurine | HMDB00251 | 107-35-7 | zwitterion |
| threonine | HMDB00167 | 72-19-5 | amino acid zwitterion |
| tryptophan | HMDB00929 | 73-22-3 | amino acid zwitterion |
| tyrosine | HMDB00158 | 60-18-4 | aromatic amino acid |
| valine | HMDB00883 | 72-18-4 | branched-chain amino acid |
